# Risk of Major Depression Associated with Excessive Daytime Sleepiness in Apneic Individuals

**DOI:** 10.3390/clockssleep7020022

**Published:** 2025-04-30

**Authors:** Matteo Conenna, Camille Point, Benjamin Wacquier, Jean-Pol Lanquart, Matthieu Hein

**Affiliations:** 1 Azienda Ospedaliera Universitaria “Luigi Vanvitelli”, 80138 Naples, Italy; matteo.conenna@gmail.com; 2 Faculté de Médecine, Université Libre de Bruxelles (ULB), 1070 Brussels, Belgium; camille.point@ulb.be; 3 Hôpital Universitaire de Bruxelles, Service de Psychiatrie et Laboratoire du Sommeil, Université Libre de Bruxelles (ULB), 1070 Brussels, Belgium; benjamin.wacquier@hubruxelles.be; 4 Laboratoire de Recherches Psychiatriques (ULB266), Université Libre de Bruxelles (ULB), 1070 Brussels, Belgium; secmed.psy@erasme.ulb.ac.be; 5 Laboratoire de Psychologie Médicale et Addictologie (ULB312), CHU Brugmann, Université Libre de Bruxelles (ULB), 1020 Brussels, Belgium

**Keywords:** excessive daytime sleepiness, major depressive disorder, obstructive sleep apnea syndrome, epidemiology, risk factor

## Abstract

Considering the frequent co-occurrence of major depressive disorder and excessive daytime sleepiness in apneic individuals, this study aimed to explore the relationship between excessive daytime sleepiness and the risk of developing major depressive disorder in this specific subpopulation. Demographic and polysomnographic data were retrospectively extracted from the clinical database of 1849 apneic individuals at the Sleep Unit. Excessive daytime sleepiness was considered present when the Epworth Sleepiness Scale score was >10 and major depressive episodes were diagnosed according to DSM criteria. Logistic regression analyses were performed to assess the risk of major depressive disorder associated with excessive daytime sleepiness in apneic individuals. The prevalence of major depressive disorder was 26.3% in apneic individuals. After controlling for major confounding variables, multivariate logistic regression analyses revealed that apneic individuals with complaints of excessive daytime sleepiness had a higher likelihood of developing major depressive disorder compared to those without complaint of excessive daytime sleepiness. This study highlights the strong association between excessive daytime sleepiness and major depressive disorder in apneic individuals, underlining the importance of systematically assessing and adequately treating excessive daytime sleepiness to better manage depressive symptoms and improve overall treatment outcomes in this specific subpopulation.

## 1. Introduction

Based on available data, a particular relationship seems to exist between obstructive sleep apnea syndrome (OSAS) and major depressive disorder (MDD). Indeed, the prevalence of MDD is high among apneic individuals, whereas OSAS is a frequent comorbidity among major depressed individuals [1,2]. In addition, OSAS is associated with a higher risk of developing a major depressive episode, and MDD appears to promote the occurrence of OSAS [3,4]. Furthermore, the presence of comorbid MDD in apneic individuals appears to be associated with a more frequent occurrence of cardiometabolic pathologies, a more pronounced deterioration in life quality, an increased risk of road accidents, and higher mortality [5,6,7,8,9]. From a therapeutic perspective, there is evidence supporting a more complex management of both MDD and OSAS in the case of co-occurrence of these two disorders. Indeed, in major depressed individuals, the presence of comorbid OSAS seems to be associated with resistance to antidepressant treatments, whereas the presence of comorbid MDD may lead to lower compliance and adherence to OSAS treatments in apneic individuals [10,11,12]. Thus, given these various elements, a better identification of factors involved in the occurrence of MDD in apneic individuals seems necessary to open new perspectives for the management of this psychiatric disorder in this particular subpopulation.

Excessive daytime sleepiness (EDS) is one of the most common sleep-related patient symptoms that is characterized by a higher risk of road/work accident and a negative impact on health status [13]. In the literature, there are arguments supporting interplay between MDD and EDS: EDS complaints are frequent among major depressed individuals, and MDD is a frequent condition among sleepy individuals [14,15]. Moreover, MDD is associated with a higher risk of EDS, whereas EDS may promote the development of major depressive episodes [16,17]. Furthermore, the severity of EDS complaints seems to be correlated with the severity of depressive symptoms [18]. However, although EDS is frequent among apneic individuals [19], the potential role played by this symptom in the occurrence of major depressive episodes has been poorly studied in this particular subpopulation [20,21,22]. Additionally, all available studies have mainly focused on the involvement of EDS in the occurrence of depressive symptoms measured by self-questionnaire in apneic individuals, which does not allow for the generalization of their results to MDD diagnosed during a systematic psychiatric interview [20,21,22]. Given this major limitation of the available studies, it could be interesting to study the risk of MDD associated with EDS complaints in apneic individuals in order to enable better identification of individuals at risk of MDD in this particular subpopulation.

For this study, the primary objective was to investigate the risk of MDD associated with EDS complaints in apneic individuals, and the secondary objective was to investigate the prevalence of MDD in this specific subpopulation. Our primary hypothesis was that EDS complaints are associated with a higher risk of MDD in apneic individuals, whereas our secondary hypothesis was that MDD is a frequent comorbidity in this particular subpopulation. The aim of this approach was to provide healthcare professionals with reliable data regarding the risk of MDD associated with EDS complaints and the prevalence of this psychiatric disorder in apneic individuals in order to enable better prevention of the potential negative impact of this psychiatric disorder in this particular subpopulation.

## 2. Results

### 2.1. Polysomnographic Data (Table 1)

Compared to apneic individuals without MDD, apneic individuals with MDD presented:

1. An increase in sleep latency (42.5 [20.3–82.5] vs. 27.0 [14.0–58.0], *p* < 0.001), REM latency (92.5 [64.5–163.5] vs. 83.5 [60.3–125.3], *p* < 0.001) and % stage 3 (6.0 [0.5–14.4] vs. 3.5 [0.3–9.9], *p* < 0.001) (Table 1).
2. A reduction in sleep efficiency (75.2 [64.1–83.5] vs. 76.5 [66.1–84.4], *p* = 0.017), sleep period time (439.5 [401.0–481.0] vs. 453.0 [411.5–484.5], *p* = 0.007), % stage 1 (8.3 [5.4–11.5] vs. 8.6 [5.4–11.5], *p* = 0.036), % REM sleep (14.6 [10.0–19.5] vs. 15.6 [11.2–19.8], *p* = 0.048), number of awakenings (29 [20,21,22,23,24,25,26,27,28,29,30,31,32,33,34,35,36,37,38,39,40,41,42,43,44] vs. 33 [22,23,24,25,26,27,28,29,30,31,32,33,34,35,36,37,38,39,40,41,42,43,44,45,46,47,48], *p* = 0.001), micro-arousal index (13 [8,9,10,11,12,13,14,15,16,17,18,19,20,21,22] vs. 14 [9,10,11,12,13,14,15,16,17,18,19,20,21,22,23,24], *p* = 0.019), apnea-hypopnea index (14 [8,9,10,11,12,13,14,15,16,17,18,19,20,21,22,23,24,25,26,27] vs. 16 [8,9,10,11,12,13,14,15,16,17,18,19,20,21,22,23,24,25,26,27,28,29,30,31,32,33], *p* = 0.015), and total time under 90% of oxygen saturation (7.5 [0.5–45.0] vs. 10.5 [1.0–58.5], *p* = 0.021) (Table 1).

There were no significant differences for other polysomnographic variables between the two groups (Table 1).

### 2.2. Univariate Analyses (Table 2)

MDD was present in 26.3% of apneic individuals in our sample (Table 2). For apneic individuals included in this study, male gender (OR 0.39 [95% CI 0.31–0.49], *p* < 0.001), age ≥ 50 and < 65 years (OR 0.71 [95% CI 0.57–0.88], *p* = 0.002), age ≥ 65 years (OR 0.56 [95% CI 0.38–0.82], *p* = 0.002) and severe OSAS (OR 0.71 [95% CI 0.55–0.92], *p* = 0.010) were significantly associated with lower risk of MDD whereas obesity (OR 1.50 [95% CI 1.22–1.84], *p* < 0.001), use of antidepressants (OR 4.59 [95% CI 3.57–5.91], *p* < 0.001), taking benzodiazepine receptor agonists (OR 3.14 [95% CI 2.29–4.32], *p* < 0.001), use of other psychotropic drugs (OR 2.74 [95% CI 1.92–3.92], *p* < 0.001), insomnia without short sleep duration (OR 4.31 [95% CI 3.24–5.73], *p* < 0.001), insomnia with short sleep duration (OR 4.52 [95% CI 3.34–6.11], *p* < 0.001), CRP levels ≥ 3 mg/L (OR 1.29 [95% CI 1.04–1.61], *p* = 0.022) and EDS complaints (OR 2.07 [95% CI 1.68–2.55], *p* < 0.001) were significantly associated with higher risk of MDD (Table 2). Furthermore, apneic individuals with MDD had younger age (49 [39,40,41,42,43,44,45,46,47,48,49,50,51,52,53,54,55,56] vs. 52 [43,44,45,46,47,48,49,50,51,52,53,54,55,56,57,58,59,60], *p* < 0.001) and higher BMI (30.1 [26.1–35.1] vs. 28.7 [25.7–32.7], *p* < 0.001)/Epworth Sleepiness Scale scores (11 [7,8,9,10,11,12,13,14,15] vs. 9 [5,6,7,8,9,10,11,12], *p* < 0.001)/Insomnia Severity Index scores (17 [13,14,15,16,17,18,19,20] vs. 12 [7,8,9,10,11,12,13,14,15,16], *p* < 0.001)/13-item Beck Depression Inventory scores (12 [9,10,11,12,13,14,15,16] vs. 2 [1,2,3,4], *p* < 0.001)/CRP levels (1.8 [1.0–4.2] vs. 1.6 [0.9–3.4], *p* = 0.004) than those without MDD (Table 2). There were no significant differences for other demographic variables between the two groups (Table 2). Finally, in our sample, EDS complaints were present in 40.1% of apneic individuals (Table 2).

### 2.3. Multivariate Analyses (Table 3)

After hierarchically introducing the significant confounders highlighted during the univariate analyses for adjustment, multivariate logistic regression models demonstrated that EDS complaints were associated with a higher risk of MDD (OR 1.65 [95% CI 1.30–2.10], *p* < 0.001) in apneic individuals (Table 3).

### 2.4. Additional Multivariate Analyses for Apneic Individuals Without Psychotropic Treatments (Table 4)

After adjusting for the main confounding factors demonstrated in the univariate analyses, additional multivariate logistic regression analyses have highlighted that EDS complaints were still significantly associated with higher risk of MDD (OR 1.78 [95% CI 1.33–2.38], *p* < 0.001) in apneic individuals without psychotropic medications (Table 4).

### 2.5. Additional Multivariate Analyses for Apneic Individuals Without Comorbid Insomnia (Table 5)

After adjustment for the main confounding factors identified during the univariate analyses, additional multivariate logistic regression analyses confirmed that EDS complaints remained significantly associated with higher risk of MDD (OR 1.97 [95% CI 1.35–2.88], *p* < 0.001) in apneic individuals without comorbid insomnia (Table 5).

## 3. Discussion

Compared to the general population [23], we have highlighted that MDD is a more frequent problem in apneic individuals (26.3% vs. 9.2%). However, this prevalence of MDD demonstrated in our study seems to be lower than that of the studies by Qin et al. (2024) (34.4%) and by Gharsalli et al. (2022) (35.0%), which could be explained by differences in the method used for the diagnosis of MDD [24,25]. Indeed, in these two studies, the use of self-questionnaires for the diagnosis of MDD may have favored an overestimation of the prevalence of this psychiatric disorder in apneic individuals since the self-questionnaires were not developed to diagnose MDD but only to screen for the presence of depressive symptoms [26,27]. On the other hand, this prevalence of MDD highlighted in our study seems to be higher than that of the studies by Lee et al. (2023) (15.5%) and by Li et al. (2023) (13.7%), which could be explained by differences in the recruitment of apneic individuals [28,29]. Indeed, in the study by Lee et al. (2023), the apneic individuals recruited presented more severe OSAS than in our study, whereas in the study by Li et al. (2023), the presence of OSAS was self-reported via a questionnaire on sleep disorders [28,29]. However, in the literature, there are arguments in favor of a reduction in the prevalence of MDD with OSAS severity and an increased risk of recruiting individuals without OSAS when using screening questionnaires in the general population [30,31], which may have led to an underestimation of the prevalence of MDD in these two studies. Finally, this prevalence of MDD demonstrated in our study seems to be similar to that of the studies by Hein et al. (2024) (23.9%) and by Jackson et al. (2019) (23.0%), in which MDD was diagnosed by a systematic psychiatric interview similar to our methodology [32,33]. Thus, given this high prevalence of MDD regardless of these methodological differences in the available studies, it seems essential to systematically screen for major depressive episodes through a psychiatric interview in apneic individuals to avoid the negative consequences related to this psychiatric disorder in this particular subpopulation.

Consistent with literature data [34,35,36], we have highlighted that EDS is a frequent complaint in apneic individuals. Indeed, the prevalence of EDS complaints was 40.1% in our sample of apneic individuals. However, some factors could help to better understand this high prevalence of EDS complaints in apneic individuals. Indeed, in apneic individuals, intermittent hypoxia and excessive sleep fragmentation related to obstructive events may induce the occurrence of alterations in sleep/wake patterns through alterations in sleep architecture (decrease in stage 3 and REM sleep—increase in stage 2 and stage 1) and damage in brain regions promoting wakefulness (oxidative injury, cell loss, and neuronal damage) [37,38]. However, the existence of these alterations of sleep/wake patterns induced by this negative synergistic effect of intermittent hypoxia and excessive sleep fragmentation associated with OSAS is one of the main pathophysiological mechanisms involved in the development of EDS for apneic individuals [37,38].

On the other hand, we demonstrated that EDS complaints were associated with a more frequent occurrence of MDD in apneic individuals, which could be explained by several specific elements. First, there is evidence supporting the involvement of EDS complaints in the occurrence of poorer life quality and worse functional status for apneic individuals [39,40]. However, it has been shown that following their negative impacts on psychological functioning, the presence of these alterations in life quality and functional status may promote the development of MDD [41]. Second, in apneic individuals with EDS complaints, the existence of some specific sleep patterns (sleep deprivation, excessive sleep fragmentation, and intermittent hypoxia) may induce the occurrence of alterations in circadian rhythms through modification of melatonin and cortisol secretion [42,43,44]. Nevertheless, the available data seem to indicate that the occurrence of these alterations of circadian rhythms plays a central role in the pathophysiology of MDD given their deleterious effects on mood regulation [45,46]. Third, alterations in daytime functioning induced by OSAS may induce a reduction in physical activity, promoting weight gain in apneic individuals [47,48]. However, obesity may promote the development of MDD following the activation of pathophysiological mechanisms (inflammation, insulin/leptin resistance, and hypertension) with a negative impact on the neuroimmune status and the neural circuits controlling mood/emotional states [49]. Thus, given their high prevalence and their potential involvement in the development of MDD, it seems essential to systematically screen and adequately treat EDS complaints in apneic individuals in order to avoid their potential negative consequences on mental health in this particular subpopulation.

Emphasizing the potential role of EDS complaints in the development of MDD among apneic individuals could lead to new therapeutic strategies for managing and preventing this psychiatric disorder in this specific population. Nevertheless, before introducing treatments specifically targeting EDS complaints, it is necessary to adequately treat OSAS [50,51]. Indeed, in apneic individuals, it has been shown that conventional treatments for OSAS (continuous positive airway pressure and mandibular advancement device) were associated with a reduction in both EDS complaints and depressive symptoms [52,53]. In addition, in parallel with these conventional treatments for OSAS, measures targeting diet, alcohol consumption, smoking, physical activity, and overweight/obesity are essential in apneic individuals given the potential positive impact of a healthy lifestyle on EDS complaints and depressive symptoms in this specific subpopulation [54]. However, despite adequate management of OSAS, some apneic individuals may potentially have residual EDS complaints that may promote the development or maintenance of depressive symptoms [55,56]. Regarding these residual EDS complaints in treated apneic individuals, several therapeutic strategies are currently recommended: adaptation of treatment for OSAS, improvement of sleep hygiene, treatment of comorbidities, elimination of iatrogenic causes, and specific drug treatments [57,58]. Among these drug options, modafinil (norepinephrine and dopamine reuptake blocker), solriamfetol (selective dopamine and norepinephrine reuptake inhibitor), and pitolisant (selective histamine H3-receptor antagonist) may be used for the treatment of residual EDS complaints in treated apneic individuals given their wake-promoting properties [57,58]. However, although modafinil is recommended as an add-on therapy for MDD due to its positive effect on mood [59], data for solriamfetol and pitolisant regarding their potential effect on depressive symptoms are currently limited [60,61], which justifies cautious use of these two molecules in patients with current or past MDD [62]. Finally, in addition to these therapeutic strategies targeting EDS complaints, it is essential to follow treatment recommendations in cases of comorbid MDD for apneic individuals in order to avoid the persistence of depressive symptoms in this specific subpopulation [63]. Nevertheless, in the case of prescription of pharmacological treatments for MDD in apneic individuals, it will be necessary to take into account their potential side effects to avoid the recurrence or worsening of EDS complaints. Indeed, in individuals with MDD, the use of some non-sedative classes of antidepressants (SSRIs or SNRIs) without combined hypnotic treatment may potentially induce the occurrence of EDS complaints due to their negative impact on sleep architecture, whereas the appropriate use of some sedative antidepressants or benzodiazepine receptor agonists with short or intermediate half-lives may be associated with improved daytime functioning due to their beneficial effect on sleep complaints [14,35,64,65,66]. However, our additional multivariate analyses highlighted that after excluding the 462 apneic individuals with psychotropic treatments (25% of the total sample), EDS complaints remained significantly associated with higher risk of MDD, which seems to indicate that this particular relationship between MDD and EDS complaints in apneic individuals exists independently of potential use of psychotropic treatments.

Alongside this potential impact of EDS complaints, several other factors that could play a major role in the occurrence of MDD in apneic individuals were highlighted during our univariate analyses. Indeed, in our sample of apneic individuals, some demographic factors (female gender, younger age, and obesity) were associated with higher risk of MDD, which seems consistent with the literature available for this specific subpopulation [67]. Regarding this impact of gender on the risk of MDD, one of the main explanations could be the existence of a vulnerability to mood disorders in women induced by the existence of recurrent hormonal fluctuations secondary to genetic and physiological factors [68,69]. For this higher risk of MDD highlighted in younger individuals, several elements may help to better understand this more frequent occurrence of this psychiatric disorder in this age group [70]. Indeed, compared to older individuals, younger individuals are generally more exposed to some factors (multiple psychosocial stressors, severe acute stressors, chronic social adversity, substance use disorders, and deleterious lifestyle factors) that may promote a more frequent occurrence of major depressive episodes following their central role in the pathophysiology of this psychiatric disorder [70]. Regarding this effect of obesity on the risk of MDD, it appears to be mediated by several specific factors since in obese individuals, the more frequent development of MDD could be explained by poor diet, sedentary lifestyle, and accumulation of visceral fat favoring the activation of some deleterious pathophysiological mechanisms (inflammation, insulin/leptin resistance, and hypertension) for the regulation of mood and emotions [49,71]. On the other hand, in our sample of apneic individuals, some sleep-related factors (insomnia disorder and OSAS severity) also appeared to play a role in the risk of developing MDD. Indeed, the presence of insomnia disorders with or without short sleep duration was associated with a higher risk of MDD, which could be explained by the existence of common pathophysiological mechanisms between these two disorders [72,73,74,75,76]. Among these common pathophysiological mechanisms, the phenomenon of hyperarousal (hypervigilance state present throughout the 24 h cycle) is currently one of the theories proposed to explain this higher propensity to develop MDD in individuals with insomnia disorder [72,73,74,75,76]. Regarding the OSAS severity, we highlighted in this study that severe OSAS was associated with a lower risk of MDD, which seems to be contradictory with current literature [77]. However, in the majority of these available studies [77], MDD was diagnosed with self-administered questionnaires, unlike our study, where MDD was diagnosed during a semi-structured psychiatric interview. However, given the existence of a symptomatic overlap between OSAS and MDD [78], this use of self-administered questionnaires rather than semi-structured psychiatric interviews may have led to overdiagnosis of MDD in individuals with severe OSAS in these studies. Furthermore, based on animal data and on the analyses of the polysomnographic data of this study, there appears to be evidence supporting a protective role of recurrent intermittent hypoxia against the development of mood disorders [79,80]. Indeed, this recurrent intermittent hypoxia seems to be associated with hippocampal neurogenesis, promoting an antidepressant-like effect [79,80], which could potentially explain this protective effect of severe OSAS against MDD highlighted in our study. Finally, in our sample of apneic individuals, some biological factors (CRP levels ≥3 mg/L) were associated with a higher risk of MDD. Indeed, consistent with the literature [81,82,83], we confirmed that the presence of low-grade inflammation played a central role in the occurrence of MDD, which could be explained by the potential negative impact of this low-grade inflammation on the central synthesis and reuptake of monoamine neurotransmitters (dopamine, serotine, and noradrenaline) [84]. Thus, based on these different elements, it is essential to carry out a holistic assessment of apneic individuals in order to enable better prevention and adequate treatment of MDD in this particular subpopulation.

Regarding polysomnographic parameters, several differences were demonstrated between apneic individuals with and without MDD in this study. Consistent with the literature [73,74,85,86,87], our analyses confirmed that individuals with MDD from our sample presented an increase in sleep latency and a decrease in sleep efficiency and sleep period time. However, in contradiction with the available data for polysomnographic parameters in major depressed individuals [73,74,85,86,87], we demonstrated that individuals with MDD from our sample presented an increase in % stage 3 and REM latency as well as a decrease in % stage 1, % REM sleep, number of awakenings, and micro-arousal index. However, these differences compared to the literature could be explained by the fact that in our sample, the use of psychotropic treatments was not an exclusion criterion. Indeed, some psychotropic treatments may have a major impact on sleep architecture (REM suppressive effect for some antidepressants, reduction in sleep fragmentation for some hypnotics, and increase in % stage 3 for some sedative antidepressants) [88,89,90,91]. In this context, it seems essential to be cautious in the interpretation of our analyses concerning polysomnographic parameters, given the potential impact of the use of psychotropic treatments in our sample of apneic individuals on sleep architecture.

### Limitations

Since the data used in this study were retrospectively extracted from the Sleep Unit database of the University Hospital of Brussels without direct validation from the apneic individuals selected, additional prospective studies are essential to confirm the findings obtained from our analyses. One limitation of this study is the reliance on a subjective measure for assessing excessive daytime sleepiness, namely the Epworth Sleepiness Scale. While the Epworth Sleepiness Scale is widely accepted in both clinical and research contexts, it depends on self-reported data, which may be influenced by personal perception and bias. This introduces variability that may impact the accuracy of the results. As such, it would be beneficial to complement or replace this subjective tool with more objective measures in order to achieve a more robust and accurate assessment of daytime sleepiness in future research. Lastly, it is important to note that the Sleep Unit database at the University Hospital of Brussels includes only apneic individuals who agreed to undergo polysomnographic recordings, which may have introduced a recruitment bias in this study.

## 4. Materials and Methods

### 4.1. Population

1849 apneic individuals were retrospectively selected from the database of polysomnographic recordings carried out between 01/01/2002 and 31/10/2023 within the Sleep Unit of the Brussels University Hospital. The inclusion criteria for this study were the presence of OSAS according to the diagnostic criteria of the American Academy of Sleep Medicine (obstructive apnea–hypopnea index ≥ 5/h) [92], the absence of acute and/or uncontrolled infectious or somatic diseases, the absence of some sleep disorders (central hypersomnia, predominantly central sleep apnea syndrome and parasomnia), the absence of previous OSAS treatment, the absence of acquired or congenital brain damage and the absence of thoracic or orofacial malformations whereas the exclusion criteria for this study were an age <18 years, the presence of psychiatric disorders other than MDD, the presence of past or current substance use disorders and the presence of pregnancy. Furthermore, in this study, we decided to focus recruitment only on apneic individuals since the main objective was to determine the role played by EDS complaints in the occurrence of MDD for this particular subpopulation. Finally, the care pathway of these apneic individuals from their first outpatient consultation in sleep medicine until their admission to the Sleep Unit is available in the Supplementary Materials—Annex S1.

### 4.2. Methods

#### 4.2.1. Medical and Psychiatric Check-Up of Apneic Individuals

A systematic medical interview and a complete somatic assessment (physical examination, blood tests, electrocardiogram, electroencephalogram, and urine analyses) were carried out by a physician on all these apneic individuals admitted to the Sleep Unit in order to diagnose all their potential medical comorbidities.

Subsequently, a semi-structured psychiatric interview was conducted by a psychiatrist assigned to the Sleep Unit in all these apneic individuals to diagnose their potential comorbid psychiatric disorders according to DSM-IV-TR (before 2013) and DSM-5 (after 2013) diagnostic criteria [93,94]. Following this semi-structured psychiatric interview, current major depressive episodes were defined as the presence of significant symptoms or signs of MDD according to DSM-IV-TR (before 2013) and DSM-5 (after 2013) diagnostic criteria for a period of at least 2 weeks before the polysomnographic recording [93,94].

Finally, all these apneic individuals benefited from a standardized assessment of their subjective complaints of depression, insomnia, and daytime sleepiness through the use of self-questionnaires described in Supplementary Materials—Annex S2 (Beck Depression Inventory [BDI reduced to 13 items], Insomnia Severity Index, and Epworth Sleepiness Scale) [95,96,97]. Thus, based on this series of self-questionnaires, EDS complaints were therefore considered to be present in apneic individuals recruited for this study when the Epworth Sleepiness Scale score was >10 [97].

#### 4.2.2. Sleep Investigation of Apneic Individuals

In all these apneic individuals, a specific sleep interview targeting sleep habits and sleep-related complaints was systematically carried out by a physician assigned to the Sleep Unit in order to identify the presence of potential signs or symptoms suggestive of the main sleep disorders. Subsequently, in compliance with the conditions of hospitalization in the Sleep Unit (Supplementary Materials—Annex S3), all these apneic individuals benefited from polysomnographic recordings with a montage meeting the criteria of the American Academy of Sleep Medicine (Supplementary Materials—Annex S4) [98]. Regarding the interpretation of these polysomnographic recordings, a visual scoring meeting international recommendations (Supplementary Materials—Annex S5) was carried out by specialized technicians under the supervision of certified somnologists to enable the production of technical reports [99,100,101]. The polysomnographic parameters extracted from these technical reports for this study were sleep latency, sleep efficiency, sleep period time, total sleep time, % stage 1, % stage 2, % stage 3, % REM sleep, REM latency, % wake after sleep onset, number of awakenings, micro-arousal index, obstructive apnea–hypopnea index, oxygen desaturation index, total time under 90% of oxygen saturation, and periodic limb movements index. Finally, thanks to this specific sleep interview and these technical reports of polysomnographic recordings, the physicians assigned to the Sleep Unit were able to confirm the diagnosis of OSAS suspected during the outpatient care pathway and systematically screen for potential comorbid sleep disorders in all these apneic individuals (diagnostic criteria for sleep disorders available in Table 6) [102,103,104,105,106].

### 4.3. Statistical Analyses

Stata software version 14 was used for statistical analyses. To enable these analyses, our sample of apneic individuals was divided into two groups: a group without MDD and a group with MDD. Only individuals with a major depressive episode meeting the DSM-IV-TR (before 2013) and DSM-5 (after 2013) diagnostic criteria were included in the group with MDD [93,94].

Since parametric tests were not usable for most of the continuous data in this study after checking their distribution and equality of variances, we decided to use non-parametric tests: medians and their P25–P75 were used for descriptive analyses, whereas Wilcoxon tests were used for comparison analyses. For categorical data, percentages were used for descriptive analyses, and Chi^2^ tests were used for comparison analyses.

Univariate logistic regression models were used to study the risk of MDD associated with EDS complaints and potential confounders (Supplementary Materials—Annex S6) [30,33,67,107,108,109,110,111,112]. Subsequently, in multivariate logistic regression models, this risk of MDD associated with EDS complaints was adjusted through a hierarchical introduction of significant confounders identified during univariate analyses. Finally, the validity of the final logistic regression model (adequacy and specificity) was checked using the Hosmer and Lemeshow test and the Link test.

Results were considered significant when the *p*-value was <0.05.

## 5. Conclusions

In this study, we confirmed that MDD is a frequent comorbidity (26.3%) in apneic individuals. Furthermore, we demonstrated that EDS complaints were associated with a higher risk of MDD in this specific subpopulation, which seems to justify systematic screening and adequate treatment of this central symptom of OSAS in order to avoid its potential negative consequences on mental health in apneic individuals. Finally, future prospective studies should be carried out to validate these findings highlighted in this study.

## Figures and Tables

**Table 1:** Polysomnographic data (n = 1849).

	Whole Sample (n = 1849)	Subjects Without MDD (n = 1362)	Subjects with MDD (n = 487)	*p*-Value
Sleep latency (min)	30.5 (15.0–64.5)	27 (14.0–58.0)	42.5 (20.3–82.5)	<0.001
Sleep efficiency (%)	76.2 (65.9–84.0)	76.5 (66.1–84.4)	75.2 (64.1–83.5)	0.017
Sleep period time (min)	450.5 (409.3–483.5)	453 (411.5–484.5)	439.5 (401.0–481.0)	0.007
Total sleep time (min)	378 (331.5–423.5)	377.8 (332.3–424.0)	378.5 (327.3–418.5)	0.195
% stage 1	8.5 (5.7–12.3)	8.6 (5.8–12.6)	8.3 (5.4–11.5)	0.036
% stage 2	53 (46.1–59.2)	53.3 (46.7–59.2)	52.2 (44.3–59.4)	0.081
% stage 3	3.9 (0.4–11.0)	3.5 (0.3–9.9)	6 (0.5–14.4)	<0.001
% REM sleep	15.3 (10.9–19.7)	15.6 (11.2–19.8)	14.6 (10.0–19.5)	0.048
REM latency (min)	85 (61.0–132.5)	83.5 (60.3–125.3)	92.5 (64.5–163.5)	<0.001
% WASO	13.4 (7.6–21.9)	13.7 (7.9–22.1)	12.5 (6.5–21.1)	0.097
Number of awakenings	32 (22–47)	33 (22–48)	29 (20–44)	0.001
Micro-arousal index	14 (9–23)	14 (9–24)	13 (8–22)	0.019
Apnea–hypopnea index	15 (8–31)	16 (8–33)	14 (8–27)	0.015
Oxygen desaturation index	8 (2–19)	8 (2–19)	7 (3–19)	0.854
Total time under 90% of SaO_2_ (min)	9 (1.0–54.0)	10.5 (1.0–58.5)	7.5 (0.5–45.0)	0.021
PLMS index	1 (0–9)	1 (0–9)	1 (0–7)	0.172
	Median (P25–P75)	Median (P25–P75)	Median (P25–P75)	Wilcoxon test

MDD = major depressive disease, REM = rapid eye movement sleep, WASO = wake after sleep onset, SaO_2_ = oxygen saturation, PLMS = periodic limb movements during sleep.

**Table 2:** Univariate analyses (n = 1849).

Variables	Categories	%	Subjects Without MDD	Subjects with MDD	*p*-Value Chi^2^	OR (CI 95%)	*p*-Value
Gender	Female (n = 483)	26.1%	21.0%	40.5%	<0.001	1	<0.001
	male (n = 1366)	73.9%	79.0%	59.5%		0.39 (0.31 to 0.49)	
Age (years)	<50 (n = 850)	46.0%	43.4%	53.2%	0.001	1	<0.001
	≥50 and <65 (n = 796)	43.0%	44.6%	38.6%		0.71 (0.57 to 0.88)	
	≥65 (n = 203)	11.0%	12.0%	8.2%		0.56 (0.38 to 0.82)	
BMI (kg/m^2^)	<30 (n = 1047)	56.6%	59.3%	49.3%	<0.001	1	<0.001
	≥30 (n = 802)	43.4%	40.7%	50.7%		1.50 (1.22 to 1.84)	
Antidepressant therapy	No (n = 1528)	82.6%	89.2%	64.3%	<0.001	1	<0.001
	Yes (n = 321)	17.4%	10.8%	35.7%		4.59 (3.57 to 5.91)	
Benzodiazepine receptor agonists	No (n = 1676)	90.6%	93.6%	82.3%	<0.001	1	<0.001
	Yes (n = 173)	9.4%	6.4%	17.7%		3.14 (2.29 to 4.32)	
Other psychotropic drugs	No (n = 1716)	92.8%	94.9%	87.1%	<0.001	1	<0.001
	Yes (n = 133)	7.2%	5.1%	12.9%		2.74 (1.92 to 3.92)	
Smoking	No (n = 1457)	78.8%	78.9%	78.6%	0.923	1	0.923
	Yes (n = 392)	21.2%	21.1%	21.4%		1.01 (0.79 to 1.30)	
Alcohol	No (n = 1114)	60.3%	59.3%	63.0%	0.143	1	0.143
	Yes (n = 735)	39.7%	40.7%	37.0%		0.85 (0.69 to 1.06)	
Cardiometabolic comorbidities	0 (n = 388)	21.0%	20.9%	21.4%	0.444	1	0.445
	1–2 (n = 938)	50.7%	50.1%	52.6%		1.03 (0.79 to 1.34)	
	≥3 (n = 523)	28.3%	29.0%	26.0%		0.88 (0.65 to 1.18)	
OSAS	Mild (n = 882)	47.7%	46.3%	51.5%	0.034	1	0.034
	Moderate (n = 485)	26.2%	26.1%	26.7%		0.92 (0.72 to 1.18)	
	Severe (n = 482)	26.1%	27.6%	21.8%		0.71 (0.55 to 0.92)	
Insomnia disorder	No (n = 681)	36.8%	42.6%	20.5%	<0.001	1	<0.001
	Short sleep duration alone (n = 393)	21.3%	25.0%	10.9%		0.91 (0.63 to 1.30)	
	Insomnia without short sleep duration (n = 439)	23.7%	18.5%	38.4%		4.31 (3.24 to 5.73)	
	Insomnia with short sleep duration (n = 336)	18.2%	13.9%	30.2%		4.52 (3.34 to 6.11)	
Sleep movement disorders	No (n = 1494)	80.8%	80.0%	83.0%	0.159	1	0.160
	Yes (n = 355)	19.2%	20.0%	17.0%		0.82 (0.63 to 1.08)	
EDS	No (n = 1107)	59.9%	64.5%	46.8%	<0.001	1	<0.001
	Yes (n = 742)	40.1%	35.5%	53.2%		2.07 (1.68 to 2.55)	
CRP (mg/L)	<3 (n = 1287)	69.6%	71.1%	65.5%	0.022	1	0.022
	≥3 (n = 562)	30.4%	28.9%	34.5%		1.29 (1.04 to 1.61)	
Major depression	No (n = 1362)	73.7%					
	Yes (n = 487)	26.3%					
	Median (P25–P75)				Wilcoxon test		
Age (years)	51 (42–59)		52 (43–60)	49 (39–56)	<0.001		
BMI (kg/m^2^)	29 (25.8–33.2)		28.7 (25.7–32.7)	30.1 (26.1–35.1)	<0.001		
CRP (mg/L)	1.7 (0.9–3.6)		1.6 (0.9–3.4)	1.8 (1.0–4.2)	0.004		
ESS	9 (6–13)		9 (5–12)	11 (7–15)	<0.001		
ISI	13 (8–17)		12 (7–16)	17 (13–20)	<0.001		
BDI	4 (2–8)		2 (1–4)	12 (9–16)	<0.001		

MDD = major depressive disease, BMI = body mass index, OSAS = obstructive sleep apnea syndrome, EDS = excessive daytime sleepiness, CRP = C-reactive protein, ESS = Epworth sleepiness scale, ISI = insomnia severity index, BDI = Beck depression inventory.

**Table 3:** Multivariate analyses (n = 1849).

Variables	Model 1 OR Adjusted (CI 95%)	*p*-Value	Model 2 OR Adjusted (CI 95%)	*p*-Value	Model 3 OR Adjusted (CI 95%)	*p*-Value	Model 4 OR Adjusted (CI 95%)	*p*-Value
EDS		<0.001		<0.001		<0.001		<0.001
No	1		1		1		1	
Yes	1.93 (1.55 to 2.39)		1.89 (1.50 to 2.38)		1.66 (1.30 to 2.10)		1.65 (1.30 to 2.10)	

Model 1 = model adjusted for gender, age and BMI. Model 2 = model adjusted for gender, age, BMI, antidepressant therapy, benzodiazepine receptor agonists and other psychotropic drugs. Model 3 = model adjusted for gender, age, BMI, antidepressant therapy, benzodiazepine receptor agonists, other psychotropic drugs, OSAS severity, and insomnia disorder. Model 4 = model adjusted for gender, age, BMI, antidepressant therapy, benzodiazepine receptor agonists, other psychotropic drugs, OSAS severity, insomnia disorder, and CRP levels. EDS = excessive daytime sleepiness, BMI = body mass index, OSAS = obstructive sleep apnea syndrome, CRP = C-reactive protein.

**Table 4:** Additional multivariate analyses for apneic individuals without psychotropic treatments (n = 1387).

Variables	Model OR Adjusted (CI 95%)	*p*-Value
EDS		<0.001
No	1	
Yes	1.78 (1.33 to 2.38)	

Model = model adjusted for gender, age, BMI, OSAS severity, insomnia disorder and CRP levels. EDS = excessive daytime sleepiness, BMI = body mass index, OSAS = obstructive sleep apnea syndrome, CRP = C-reactive protein.

**Table 5:** Additional multivariate analyses for apneic individuals without comorbid insomnia (n = 1074).

Variables	Model OR Adjusted (CI 95%)	*p*-Value
EDS		<0.001
No	1	
Yes	1.97 (1.35 to 2.88)	

Model = model adjusted for gender, age, BMI, antidepressant therapy, benzodiazepine receptor agonists, other psychotropic drugs, OSAS severity and CRP levels. EDS = excessive daytime sleepiness, BMI = body mass index, OSAS = obstructive sleep apnea syndrome, CRP = C-reactive protein.

**Table 6:** Diagnostic criteria for sleep disorders.

Sleep Disorders	Diagnostic Criteria
Obstructive sleep apnea syndrome severity [102]	Mild (obstructive apnea–hypopnea index ≥ 5/h and <15/h) Moderate (obstructive apnea–hypopnea index ≥ 15/h and <30/h) Severe (obstructive apnea–hypopnea index ≥ 30/h)
Moderate to severe periodic limb movement syndrome [103]	Periodic limb movement index ≥ 15/h
Restless legs syndrome [104]	International Restless Legs Syndrome Study Group
Insomnia disorder [105]	American Academy of Sleep Medicine Work Group
Short sleep duration [106]	Sleep duration < 6 h

## Data Availability

The data presented in this study are available on request from the corresponding author.

## Supplementary Materials

The following supporting information can be downloaded at: https://www.mdpi.com/article/10.3390/clockssleep7020022/s1.
