# Supplementary material for: Risk of Major Depression Associated with Excessive Daytime Sleepiness in Apneic Individuals
Source: Clocks Sleep. 2025 Apr 30;7(2):22. doi: 10.3390/clockssleep7020022 (PMC12101287; doi:10.3390/clockssleep7020022)
Supplement: Supplementary file 1 [file clockssleep-07-00022-s001.zip › clockssleep-3424957-supplementary.pdf]

Supplementary Data

Annex 1

Care pathway of the apneic individuals from their first outpatient consultation in sleep medicine until their admission to Sleep Unit

These apneic individuals were referred to the sleep unit by physicians specialized in sleep medicine after an outpatient consultation during which a preliminary assessment of their complaints related to sleep, their ongoing psychotropic/somatic treatments and their somatic/psychiatric comorbidities was systematically performed in order to allow a first diagnostic hypothesis. The polysomnographic examinations were performed in these apneic individuals to allow an objective assessment of their sleep complaints and confirm the suspicion of OSAS highlighted during the outpatient assessment.

Annex 2

Self-questionnaires used

- The Beck Depression Inventory (reduced to 13 items) was used to investigate the presence of depressive symptoms. The 13 items of this scale may be scored from 0 to 3, which means that the total score may vary from 0 to 39. A final score of 0-4 indicates an absence of depressive symptoms, 5-7 mild depressive symptoms, 8-15 moderate depressive symptoms and ≥16 severe depressive symptoms.

- The Epworth Sleepiness Scale was used to investigate daytime sleepiness. The 8 items of this scale assessing sleepiness in different daytime situations may be scored from 0 to 3, which means that the total score may vary from 0 to 24. A final score greater than 10 indicates excessive daytime sleepiness.

- The Insomnia Severity Index was used to investigate the severity of insomnia complaints. The 7 items of this index may be scored from 0 to 4, which means that the total score may vary from 0 to 28. A final score of 0-7 indicates an absence of insomnia complaints, 8-14 subclinical insomnia complaints, 15-21 moderate insomnia complaints and 22-28 severe insomnia complaints.

Annex 3

Stay conditions at the Sleep Unit

The patients went to bed between 22:00 - 24:00 and got up between 6:00 - 8:00, following their usual schedule. During bedtime hours, the subjects were recumbent and the lights were turned off. Daytime naps were not permitted.

Annex 4

Applied polysomnography-montage

- Two electro-oculogram channels
- Three electroencephalogram channels
- One submental electromyogram channel
- Electrocardiogram
- Pressure cannula to detect the oro-nasal airflow
- Finger pulse-oximetry
- Microphone to record breathing sounds and snoring
- Plethysmographic inductive belts to measure thoracic and abdominal breathing
- Anterior tibialis electrodes

Annex 5

Polysomnographic scoring criteria

Obstructive apneas were scored if the decrease in air flow was ≥90% for at least 10 seconds whereas obstructive hypopneas were scored if the decrease in airflow was ≥30% for at least 10 seconds with a decrease in oxygen saturation of 3% or followed by micro-arousal. The obstructive apnea-hypopnea index corresponds to the total number of obstructive apneas and hypopneas divided by the period of sleep in hours.

Periodic limb movements were scored based on the following strict criteria: 1) duration between 0.5 to 10 seconds, 2) interval between 5 and 90 seconds from leg movement onset and 3) movements had to be part of a series of ≥4 consecutive movements meeting these criteria. Periodic limb movement index corresponds to the total number of periodic limb movements divided by period of sleep in hours.

Annex 6

Confounding factors included in the univariate analyses

Based on a literature review of risk factors for MDD in apneic individuals, the potential confounding factors included in this study were body mass index (categorized: <30 kg/m^2, ≥30 kg/m^2), age (categorized: <50 years, ≥50 & <65 years, ≥65 years), presence of cardiometabolic comorbidities (categorized: 0, 1-2, ≥3), OSAS severity (categorized: mild, moderate, severe), insomnia disorder (categorized: absent, short sleep duration alone, insomnia without short sleep duration, insomnia with short sleep duration), CRP levels (categorized: <3 mg/L, ≥3 mg/L) and as binary variables: gender, antidepressant therapy, benzodiazepine receptor agonists, other psychotropics drugs (antipsychotics + mood stabilizers), smoking, alcohol consumption and sleep movement disorders.
